# The Effect of Mechanical Ventilation on TASK-1 Expression in the Brain in a Rat Model

**DOI:** 10.1155/2017/8530352

**Published:** 2017-09-28

**Authors:** Buqi Na, Hong Zhang, Guangfa Wang, Li Dai, Guoguang Xia

**Affiliations:** ^1^Department of Respiratory and Critical Care Medicine, Beijing Jishuitan Hospital, Beijing 100035, China; ^2^Department of Respiratory and Critical Care Medicine, Peking University First Hospital, Beijing 100034, China

## Abstract

**Background and Objective:**

TWIK-related acid-sensitive potassium channel 1 (TASK-1) is closely related to respiratory central control and neuronal injury. We investigated the effect of MV on TASK-1's functions and explored the mechanism using a rat model.

**Methods:**

Male Sprague-Dawley rats were randomized to three groups: (1) high tidal volume (HVt): MV for four hours with Vt at 10 mL/kg; (2) low Vt (LVt): MV for four hours with Vt at 5 mL/kg; (3) basal (BAS): anesthetized and unventilated animals. We measured lung histology and plasma and brain levels of proteins (IL-6, TNF-*α*, and S-100B) and determined TASK-1 levels in rat brainstems as a marker of respiratory centre activity.

**Results:**

The LISs (lung injury scores) were significantly higher in the HVt group. Brain inflammatory cytokines levels were different to those in serum. TASK-1 levels were significantly lower in the MV groups (*P* = 0.002) and the HVt group tended to have a lower level of TASK-1 than the LVt group.

**Conclusion:**

MV causes not only lung injury, but also brain injury. MV affects the regulation of the respiratory centre, perhaps causing damage to it. Inflammation is probably not the main mechanism of ventilator-related brain injury.

## 1. Introduction

Although mechanical ventilation (MV) is often a lifesaving method in critically ill patients, it is associated with many potential complications. MV causes not only ventilation-induced lung injury, but also distal organ dysfunction [1, 2], including the brain. Acute respiratory distress syndrome (ARDS) patients may develop non-muscle related ventilator-dependence, which is probably induced by ventilation. Long-term survivors of acute lung injury (ALI)/ARDS often have significant neurocognitive and emotional morbidity, while brain injury patients are more prone to lung complications [3–5]. Thus, the brain and lung have an integrity of function. It is important to explore the interaction between the lung and the brain in patients undergoing MV; however, studies on the impact of MV on the brain are limited and mainly focused on neonates [6, 7], and how MV influenced respiratory centre activity remains poorly understood.

K^+^ currents have a key role in setting, maintaining, and regulating membrane potential and cellular activity and K_2P_ channels are major contributors to those background currents [8]. TWIK-related acid-sensitive potassium channel 1 (TASK-1) is in this family, which produces instantaneous open-rectifier (i.e., “leak”) K^+^ currents, and plays key role in the regulation of cell membrane potential [9, 10]. It has TASK-1~5 subtypes; among them, the pK of TASK-1 is ~7.4, which is very close to the normal acid and alkaline environment of the human body [11]. They are widely expressed in the central nervous system (CNS), especially in brainstem respiratory neurons and motor neuron [9, 12], including the pre-Bötzinger complex of the medulla oblongata, which is the generator of basic respiratory rhythm [9, 11–13]. Thus, TASK-1 plays a role in respiratory rhythm generation [10, 12–14]. Inhibition of the TASK-1 leads to an increased excitability of brainstem respiratory neurons, through further depolarization of cell membrane [11], thus enhancing the respiratory drive [13, 14]. Accompanied by its unique pharmacological profile, TASK-1 plays an extensive role in various physiological regulation activities including stress and inflammation and neuroimmune responses and is implicated in processes such as neuronal apoptosis [15].

We investigated the effect of MV on the brain in a rat model using TASK-1 functions as indicators. We compared TASK-1 expression in rats ventilated with two different ventilator strategies, a high tidal volume (Vt) group and a low Vt group, and compared with basal level rats.

## 2. Methods

### 2.1. Animal Preparation

This study was approved by the Animal Ethics Committee of Peking University First Hospital. We used 24 adult male Sprague-Dawley rats weighing 350 to 400 grams, which were assigned randomly to one of three experimental groups (*n* = 8 in each group): (i) the high Vt group (HVt), ventilated with 10 mL/kg and a positive end-expiratory pressure (PEEP) of 0 cm H_2_O (ZEEP) for four hours; (ii) the low Vt group (LVt), ventilated with 5 mL/kg and ZEEP for four hours; and (iii) the basal group (BAS), unventilated animals, which were immediately exsanguinated after induction of anaesthesia. Anaesthesia was performed by intraperitoneal urethane (1.2 g/kg) and paralysis was achieved using intravenous rocuronium (1 mL/kg/h). Additional anaesthesia was given when necessary. An endotracheal tube (2.5 mm inner diameter) was inserted and tightly tied to avoid air leaks. MV group animals were connected to an HX-300 ventilator (Thai Union Technology, Chengdu, China) and ventilated for four hours. Volume-controlled ventilation was used with a respiratory rate of 60 to 70 breaths/min to yield a partial pressure of carbon dioxide in arterial blood (PaCO_2_) of 35 to 40 mm Hg at baseline. A positive end-expiratory pressure of 0 mm Hg and an inspiration : expiration ratio of 1 : 1.25 were applied. The inspiratory oxygen fraction was kept at 0.3 throughout the experiment. The left carotid artery was cannulated and connected to a pressure transducer to monitor mean arterial pressure (MAP) and heart rate (HR). The right jugular vein was cannulated for fluid infusion. Fluid replacement was provided by administration of Ringer's lactate solution 10 mL·kg^−1^·h^−1^ (or in accordance with the hemodynamics).

### 2.2. Experimental Protocol

The MAP and HR values were measured at baseline and hourly thereafter; arterial blood gases were measured at baseline, 2 h, and 4 h. Fluid management was identical in both groups. Rats were euthanized by exsanguination at the end of the four-hour period, and 8 mL of blood was collected through the arterial catheter. We stored the plasma at −80°C for protein determinations. Rat brainstems were removed and immediately frozen and stored at −80°C. Hearts and lungs were removed en bloc, and the left lung was frozen for additional pathological examination.

### 2.3. Measurements

#### 2.3.1. Histological Analysis

Left lower lobes were fixed by instillation of formalin into the airway at a pressure of 5 cm H_2_O and immersed in the same fixative. A senior pathologist, who was blinded to experimental groups, calculated the histological scores after haematoxylin-eosin (HE) staining at a magnification of ×400. Lung damage was determined using a lung injury score (LIS), based on the evaluation of alveolar oedema, haemorrhage, leukocyte infiltration, alveolar septal thickening, and alveolar inflation in each animal. Each parameter was scored from 0 to 4. Subsequently, the total LIS was calculated by adding the individual scores for each parameter, up to a maximum score of 16.

#### 2.3.2. Plasma and Brain Protein Immunoassays

Commercially available enzyme-linked immunosorbent assay (ELISA) kits (Biosource, Camarillo, CA, USA) were used to determine the levels of the following proteins in plasma/brain homogenates: tumour necrosis factor-alpha (TNF-*α*), interleukin-6 (IL-6), and S-100B. Analyses of all samples, standards, and controls were run in duplicate, following the manufacturer's recommendations.

#### 2.3.3. Western Blotting for TASK-1

Rats lower brainstems were homogenized using lysis buffer containing 30 ml tris(hydroxymethyl) aminomethane hydrochloride (pH 7.5), 250 mL sucrose, 150 mL sodium chloride, 1.0 mL dichlorodiphenyltrichloroethane, and a protease inhibitor cocktail (Sigma; St. Louis, MO, USA), buffer containing another protease inhibitor mixture and phosphatase inhibitor mixture (both from Roche Applied Science, Indianapolis, IN, USA). Supernatants were collected after centrifugation at 11,180*g* at 4°C for 10 min. The protein concentration of each sample was measured using a bicinchoninic acid assay kit (KeyGEN BioTECH, Jiangsu, China) using bovine serum albumin (BSA) as a standard. An equal amount of protein extract from each sample (50 mg) was loaded and separated on a 10% Tris-glycine SDS polyacrylamide gel and transferred electrophoretically onto a nitrocellulose membrane (Pujinkangli, Beijing, China). The membrane was blocked with 5% nonfat dry milk in Tris-buffered saline containing 0.1% Tween 20 for 1 h at 25°C and then incubated for 24 h with anti-TASK-1 antibodies (1 : 200 dilution) (Alomone Labs, Jerusalem, JRS, Israel) at 4°C. The blots were then washed and incubated with goat-anti-rabbit secondary antibodies (1 : 5,000 dilution) (Zhongshanjinqiao, Beijing, China). Visualization was performed using enhanced chemiluminescence (Visual Protein Biotechnology, Taipei, Taiwan). Protein bands were quantified (the integrated optical density value of each band was calculated and corrected to that of GAPDH) using the AlphaEaseFC image analysis software package (version 3.5, Eastman Kodak, Rochester, NY, USA).

### 2.4. Statistical Analysis

All values are expressed as the mean ± SD. Mann–Whitney nonparametric *U* tests were used to analyse differences between groups, under the supervision of an expert statistician, in the SPSS 17.0 software (Chicago, IL, USA). A *P* value < 0.013 was considered statistically significant after Bonferroni correction.

## 3. Results

### 3.1. Physiological Variables

**Figure 1:**
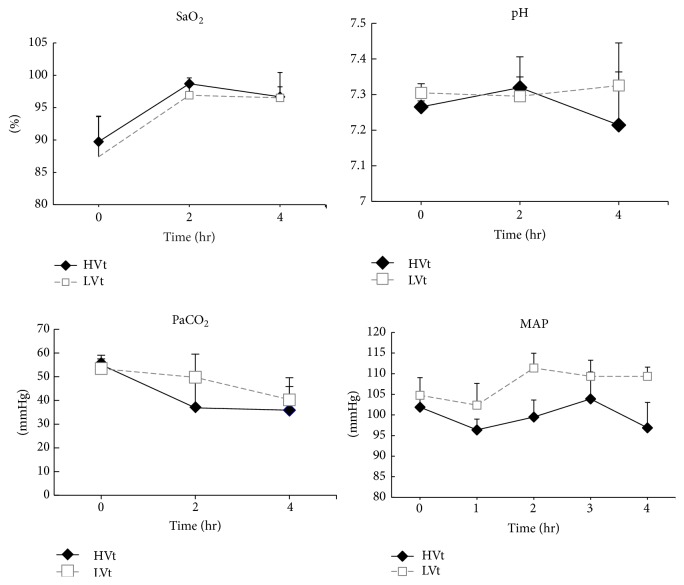
*Hemodynamic and respiratory characteristics of rats during the four-hour period of ventilation*. No differences between groups were observed at baseline. MAP remained stable in both groups. There were no differences between the LVt and HVt for arterial oxygen saturation (SaO_2_). PaCO_2_ in LVt animals was higher than in HVt animals. The pH decreased slightly in the HVt animals at four hours. But neither PaCO_2_ nor pH had significant difference between two ventilatory groups during 4-hour period. Data are presented as the mean ± SD. *N* = 8 animals per group. MAP, mean arterial pressure; BAS, basal; LVt, low tidal volume; HVt, high tidal volume; SaO_2_, arterial oxygen saturation; PaCO_2_, partial pressure of carbon dioxide in arterial blood.

MAP remained stable and within the normal range throughout the four-hour period of MV in all groups (Figure 1). Oxygen saturation was relatively low at baseline, increased after two hours, and remained stable throughout the rest of the experiment. PaCO_2_ returned to a normal range at two hours in the HVt group and remained relatively high in the LVt animals; however, no significant difference was found between the MV groups. There was a decline in the pH value in the HVt group at four hours, although it was not significant compared with that of the LVt group.

### 3.2. Histology

**Figure 2:**
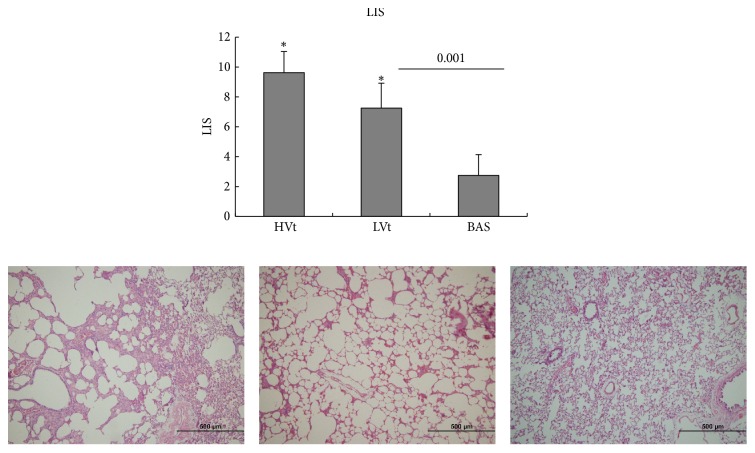
*Representative images of lungs in each group after haematoxylin and eosin staining and associated LISs*. Lung tissue of the MV group showed obvious damage, including significant alveolar oedema, haemorrhage, inflammatory cell infiltration, alveolar septal thickening, and alveolar overexpansion or atelectasis. LIS increased with MV and was significantly higher in the HVt group compared with that in the LVt group. Results are represented as the mean ± SD. ^*∗*^*P* < 0.013 versus the basal group. *N* = 8 animals per group. MV, mechanical ventilation; BAS, basal; LVt, low tidal volume; HVt, high tidal volume; LIS, lung injury score.

Figure 2 shows representative images of the lungs in rats from each experimental group. Lung tissues of the MV groups showed obvious damage. The LISs were significantly higher in the MV rats compared with that in the unventilated rats (*P* = 0.001), and the LIS of the HVt group was significantly higher than that of the LVt group (*P* = 0.001).

### 3.3. Plasma and Brain Protein Levels

**Figure 3:**
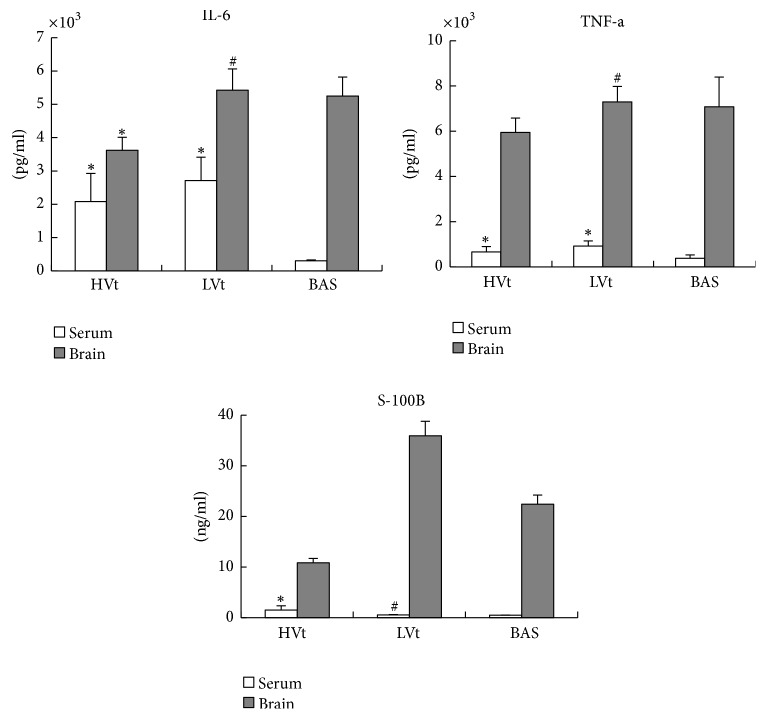
*Cytokine concentrations in plasma and brain homogenates*. MV increased plasma IL-6 and TNF-*α* significantly, irrespective of the Vt level (LVt or HVt) (*P* < 0.013). In the brain, IL-6 and TNF-*α* decreased in the HVt group. There was no difference in IL-6 and TNF-*α* levels between the LVt and basal groups. In the plasma, S-100B increased significantly in the HVt group. In brain, no correlation between S100B levels and tidal volume levels was seen. Results are presented as the mean ± SD. ^*∗*^*P* < 0.013 versus the basal group and ^#^*P* < 0.013 versus the HVt group. *N* = 8 animals per group. MV, mechanical ventilation; BAS, basal; LVt, low tidal volume; HVt, high tidal volume.

Figure 3 shows the plasma and brain levels of proinflammatory mediators. MV increased the plasma levels of IL-6 (*P* = 0.001) and TNF-*α* (HVt versus BAS *P* = 0.01, LVt versus BAS *P* = 0.001) significantly. In the brain, the HVt group showed reduced levels of IL-6 (*P* = 0.001) and TNF-*α* (*P* = 0.05) compared with the baseline level. IL-6 and TNF-*α* levels were similar in the LVt and basal groups. In the plasma, S-100B increased significantly in the HVt group (*P* = 0.001). In brain, S-100B decreased in HVt and increased in LVt group. Taken together, the inflammatory response was different in brain compared with the peripheral blood. In serum, there was higher inflammatory response in the MV groups. In the brain, LVt group showed a similar inflammatory response to the basal animals. By contrast, the HVt group exhibited a much lower inflammatory response. As for S100B, in serum, significant increase was seen in HVt group. In brain, LVt group had a higher S100B level compared to baseline, whereas there was a decrease in S100B level in HVt group.

### 3.4. TASK-1 Expression in Brainstem

**Figure 4:**
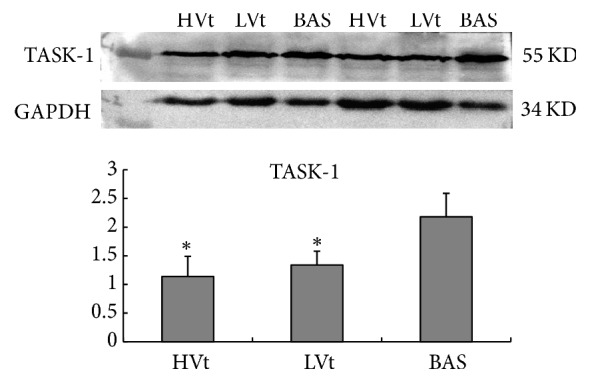
*Western blotting analysis of TASK-1 levels in each group*. Significant decreases in the TASK-1 channel levels in the HVt and LVt groups were observed. The HVt group tended to have a lower level of TASK-1 than the LVt group. Results are presented as mean ± SD. ^*∗*^*P* < 0.013 versus the basal group (HVt versus BAS *P* = 0.002, *U* = 3) (LVt versus BAS *P* = 0.002, *U* = 2). *N* = 8 animals per group. BAS, basal; LVt, low tidal volume; HVt, high tidal volume.

Figure 4 shows the TASK-1 levels in the rat brains. Differences in TASK-1 channel levels in the three groups of rats were observed. Compared with basal rats, TASK-1 channel levels were significantly lower in the HVt and LVt groups (both *P* = 0.002). In ventilated rats, the HVt group had lower levels of TASK-1 compared with that in the LVt group; however, the difference did not reach statistical significance. 

## 4. Discussion

### 4.1. Ventilation-Induced Lung Injury and Systemic Inflammation

Ventilator-associated lung injury (VILI) includes barotrauma, volutrauma, atelectrauma, and biotrauma, among which volutrauma, the overexpansion produced by high tidal volume, is the major cause of VILI [1, 16, 17]. Using histopathological analysis, we found that ventilated rats had lung injuries, with a significantly higher LIS than basal rats. Moreover, the LIS was significantly higher in the HVt group compared with that in the LVt group, indicating that MV induced lung injury, and the extent of damage increased with increasing tidal volume size, which was consistent with previous studies.

Some scholars use the concept of “organ crosstalk” to describe the effects of mechanical ventilation on multiple organs and the interactions between them [4]. However, how the lung-brain crosstalk occurs is unclear. Studies have shown that the ventilation-induced inflammatory response not only aggravates lung injury, but also leads to distal organ damage through circulation [18–21], which is the “biotrauma” mentioned above. Some researchers have suggested that inflammatory mediators in the blood can be sensed by the brain; thus, altering the permeability of the blood-brain barrier might be one possible mechanism of ventilation-related brain injury [22, 23]. However, previous studies focused on the lung and peripheral inflammation, and few addressed the inflammation status of the CNS [18, 19]. We measured IL-6 and TNF-*α* in the serum and brain and found that MV increased the blood IL-6 and TNF-*α* significantly, irrespective of the tidal volume level, whereas in the brain, a significant decrease in IL-6 was observed in the HVt group compared with that in the basal group. MV induced lung injury and the release of inflammatory cytokines into the circulation, but this systemic inflammation did not affect the brain, indicating that inflammation is not the only cause of ventilator-related brain injury.

### 4.2. Effect of MV on the Brain and TASK-1 Expression

Previous studies showed MV might promote brain activation [18] and influence cerebral tissue oxygenation and metabolism [20] and even brain tissue damage and disintegration of the blood-brain barrier [24–26]. TASK-1, is a two-pore (2P) domain potassium channel, which plays key role in the regulation of cell membrane potential [9, 10]. It is expressed widely in the CNS, especially in brainstem respiratory neurons and motor neurons [9, 27], including the pre-Bötzinger complex of the medulla oblongata, which is the generator of the basic respiratory rhythm [9, 11–13]. Therefore, TASK-1 plays a role in respiratory rhythm generation [10, 12, 14]. Inhibition of TASK-1 leads to an increased excitability of brainstem respiratory neurons, through depolarization of the cell membrane [11], thus enhancing the respiratory drive [13, 14]. Our results showed a significant decrease in TASK-1 channel levels in both the HVt and LVt groups, and there was a trend for the HVt group to have a lower level of TASK-1 compared with that in the LVt group. This suggested that MV might have an effect on respiratory centre function via changing the excitability of respiratory neurons, and this effect might correlate with the tidal volume level.

S100B is a calcium binding protein physiologically produced and released predominantly by astrocytes, because their levels may increase in CSF and blood in several brain pathologies including cell death [24–26]; it is considered to be a marker of neuronal damage. Bickenbach et al. [20] investigated the effects of different tidal volume ventilation on cerebral tissue oxygenation and metabolism in pig and found that low tidal volume (6 ml/kg) group significantly improved brain tissue oxygenation and micrometabolism compared to high tidal volume (12 ml/kg) group. In addition, serum S100B protein levels significantly increased in high tidal volume group compared to baseline level, indicating neuronal damage. In pig model of hypoxemia, Fries et al. [28] found increase of S100B in ALI pig serum, while visible damage was observed in hippocampal neurons under microscope. Also, studies showed that S100B over a certain threshold (ng/ml as a unit of measurement**)** [29, 30] are indicators of prior brain damage and bear clinical significance as predictors of poor outcome. As neurologic injury markers in blood are hampered by many confounding factors, some scholars proposed that biochemical markers in brain might be more accurate reflectors of cerebral pathological changes [24, 30]. But there are few studies comparing the prognostic value of S100B in serum and brain (CSF or brain homogenates) and the result differs [31, 32]. We for the first time measured S100B in both serum and brain during mechanical ventilation and observed that, in serum, S100B increased significantly in HVt group, which suggested neuronal damage in this group. In brain, LVt group had a higher S100B level than basal group, whereas there was a decrease in S100B level in HVt group.

MV can induce apoptosis in distant organs (including the kidney, intestine, heart, and liver) through different pathways [22]. Although many studies have speculated that potassium channels induce neuronal apoptosis [33, 34] or immune inflammatory injury [35], increasing evidence suggests that an important emerging therapeutic mechanism underlying neuroprotection is the activation/opening K_2P_ channels. Inflammatory plaques of human multiple sclerosis patients displayed profoundly lowered expression of TASK isoforms [15]. Rao et al. found that traumatic brain injury led to a downregulation of potassium channels (RK5, TWIK, and X62859) in the injured cortex, leading to decreased posttraumatic axonal conductance and epilepsy [36]. Liu et al. determined that K_2P_ channels (TASK-1, 2, 3) protected cells effectively from cytotoxic stress by preventing activation of apoptotic pathways [37]. Increased membrane excitability by TASK channel inhibition could contribute to increased electrical activity and subsequent neuronal degeneration caused by intracellular sodium and calcium accumulation, which is known as “excitotoxicity,” the most common pathological mechanism leading to neuronal death. Meuth et al. [15] observed hypoxia depolarized central neurons after specific inhibition of TASK-1; they proposed that upregulation of functional TASK channel expression might exert a neuroprotective effect by dampening neuronal excitability. Neurons expressing nitric oxide (NO) synthase (NOS-I), which is upregulated in many human chronic neurodegenerative diseases, are highly susceptible to neurodegeneration. González-Forero et al. [38] showed that the autocrine activation of the neuronal NO/cGMP pathway induced by XIIth nerve injury enhanced excitability of the motor neuron pool and fully suppressed TASK currents via a protein kinase G- (PKG-) dependent mechanism. Finally, they proposed a hypothesis whereby TASK channel inhibition via persistent autocrine activation of the NO/cGMP/PKG cascade could sensitize NOS-expressing neurons to excitotoxic damage in brain neurodegenerative processes via a sustained increase in their excitability. Moreover, Mazzone and Nistri [39] used a validated in vitro model of spinal cord injury induced by kainate-mediated excitotoxicity to explore relation of S100B levels and damage severity and found that S100B represents a useful biomarker of lesion progression as its level is related to the occurrence and severity of neuronal loss due to excitotoxicity. Therefore, MV might cause neuronal damage directly in the brain or cause apoptosis by reducing the protective factor-TASK-1 channel expression. TASK channel inhibition-mediated sensitization of neurons to excitotoxic damage might be the mechanism of the latter [36].

Our study had certain limitations: (1) as anaesthetized animals soon develop respiratory failure, a group of spontaneously breathing rats was not set in this study; thus the results did not allow us to discriminate the role played by surgical procedures. (2) We hypothesized that mechanical ventilation affects TASK-1, thereby affecting the respiratory centre activity, based on the fact that TASK1 is closely related to respiratory neuron excitability. Functional change in neuron physiology was not determined in our study. As leak conductance of TASK-1 channel under different ventilatory settings can not be measured in vivo via patch clamp techniques, a better experimental design is needed in future research to determine functional status of TASK-1 channel during MV. (3) We measured S100B as a biomarker of neuronal damage and found the change of serum S100B was consistent with literature, indicating neuronal damage in HVt, but in brain, we did not observe correlation between S100B levels and tidal volume levels. Ahmed et al. [40] had found the CSF levels of biomarkers including S100B had time-dependent changes, with first peak appearing at 6 h after the injury and second peak at 24 h or 72 h, indicating that determining S100B continuously at different time intervals might reflect neuronal injury of brain more accurately. Moreover, in subsequent studies we should detect the neuronal apoptosis by TUNEL method in frozen slices of brainstem from different groups to reflect neuronal apoptosis directly and verify the significance of serum or brain S100B in predicting neuronal damage during mechanical ventilation. (4) To explore the mechanism through which MV influenced the brain, we measured IL-6 and TNF-a in serum and brain, respectively, to reflect the difference of inflammatory response in peripheral and central nervous system and obtained some different result from previous studies [22, 23]. Taking the fact that proinflammatory cytokines IL-6 and TNF-a could be influenced by many confounding factors and blood-brain barrier as well, we would like to assess immune cells in both serum and brain during MV in subsequent studies to further reflect peripheral and central inflammatory status. Moreover, we would like to differentiate and quantify immune cells in bronchoalveolar lavage fluid of different groups in supplementary experiment to explore the effect of MV on the immunoinflammatory status of lung, peripheral blood, and CNS, respectively.

## 5. Conclusion

In conclusion, MV downregulated the TASK-1 channel level in the rat brainstem, and the effect seemed to correlate positively with the tidal volume level, indicating that MV has an effect on the respiratory centre, possibly causing damage to it by increasing neuronal excitability. We also explored the mechanism through which MV influences the brain and found that (1) MV induced lung injury, which correlated with tidal volume size; (2) MV triggered proinflammatory responses in the circulation, but this systemic inflammation did not influence the brain, which was not consistent with the broadly accepted inflammatory mechanism hypothesis.

## Abbreviations

TASK-1: TWIK-related acid-sensitive potassium channel 1
MV: Mechanical ventilation
HVt: High tidal volume
LVt: Low tidal volume
BAS: Basal
MAP: Mean arterial pressure
PaCO_2_: Partial pressure of carbon dioxide in arterial blood
LISs: Lung injury scores
ARDS: Acute respiratory distress syndrome
ALI: Acute lung injury
CNS: Central nervous system
PEEP: Positive end-expiratory pressure
HR: Heart rate
HE: Haematoxylin-eosin
TNF-*α*: Tumour necrosis factor-alpha
IL-6: Interleukin-6
VILI: Ventilator-associated lung injury
NOS-I: Nitric oxide synthase
PKG: Protein kinase G.
